# OLDER ADULTS SHARE CHALLENGES IN THEIR LIFE STORY: SENSE OF PURPOSE AS A RESOURCE FOR RESILIENCE

**DOI:** 10.1093/geroni/igac059.2164

**Published:** 2022-12-20

**Authors:** Semper Habib, Ameesha Narine, Susan Bluck, Shubam Sharma

**Affiliations:** Kennesaw State University, Kennesaw, Georgia, United States; Kennesaw State University, Kennesaw, Georgia, United States; University of Florida, Gainesville, Florida, United States; Kennesaw State University, Kennesaw, Georgia, United States

## Abstract

Maintaining a sense of purpose promotes mental and physical well-being in older adults (Musich et al., 2018). Drawing on one’s sense of purpose is thus important for late life resilience. How older adults’ sense of purpose manifests in their everyday lives remains understudied. This study used qualitative methods to amplify older adults’ voices regarding purpose and resilience through analysis of their life stories. This study 1) explored what factors contribute to maintaining purpose in older adulthood, and 2) identified how older adults draw on their purpose during major challenges, particularly in the context of the COVID-19 pandemic. Eighteen older men and women (Mage = 79.1; including the young-old, old-old, and oldest-old) participated in semi-structured life story interviews that asked about participants’ individual interpretations of purpose in their lives and their experiences navigating the COVID-19 pandemic. Thematic analysis was conducted using established methods (Braun & Clarke, 2012). To address the first research question, analyses revealed that older individuals largely maintain their purpose through engaging in acts of service to others, fostering connections with close others, and actively setting and achieving goals. Regarding the second question, older adults described how drawing on purpose through acts of service and connections with others fostered resilience through the COVID-19 pandemic. Overall, older adults’ own expressions of their life stories illuminated how they are guided by purpose. Findings demonstrate the functionality of purpose in late life and how purpose can be practically fostered, specifically within the context of universally challenging experiences such as the COVID-19 pandemic.

